# Supplementary material for: HIVprotI: an integrated web based platform for prediction and design of HIV proteins inhibitors
Source: J Cheminform. 2018 Mar 9;10:12. doi: 10.1186/s13321-018-0266-y (PMC5845081; doi:10.1186/s13321-018-0266-y)
Supplement: Supplementary file 2 — Additional file 2. Source code of HIVProtI web server. [file 13321_2018_266_MOESM2_ESM.zip › HIVprotI_Source-code/hivprotI/weka-3-6-6/doc/weka/core/neighboursearch/balltrees/BallTreeConstructor.html]

BallTreeConstructor


---


|  |  |  |
| --- | --- | --- |
| **Overview** \| **Package** \| **Class** \| **Tree** \| **Deprecated** \| **Index** \| **Help** \| **Weka's home** | |  |
| **PREV CLASS**   **NEXT CLASS** | **FRAMES**    **NO FRAMES**     **All Classes** |  |
| SUMMARY: NESTED \| FIELD \| CONSTR \| METHOD | DETAIL: FIELD \| CONSTR \| METHOD |  |


---


## weka.core.neighboursearch.balltrees Class BallTreeConstructor

```
java.lang.Object
  weka.core.neighboursearch.balltrees.BallTreeConstructor
```

**All Implemented Interfaces:**: java.io.Serializable, OptionHandler, RevisionHandler

**Direct Known Subclasses:**: BottomUpConstructor, MiddleOutConstructor, TopDownConstructor

---

``` public abstract class BallTreeConstructor extends java.lang.Object implements OptionHandler, java.io.Serializable, RevisionHandler ```

Abstract class for constructing a BallTree .

**Version:**
:   $Revision: 1.3 $

**Author:**
:   Ashraf M. Kibriya (amk14[at-the-rate]cs[dot]waikato[dot]ac[dot]nz)

**See Also:**: Serialized Form

---

**Constructor Summary**

`BallTreeConstructor()`
  
          Creates a new instance of BallTreeConstructor.


| **Method Summary** | |
| --- | --- |
| `abstract  int[]` | `addInstance(BallNode node, Instance inst)`             Adds an instance to the ball tree. |
| `abstract  BallNode` | `buildTree()`             Builds the ball tree. |
| `java.lang.String` | `containChildBallsTipText()`             Returns the tip text for this property. |
| `boolean` | `getContainChildBalls()`             Gets whether if a parent ball should completely enclose its two child balls. |
| `int` | `getMaxDepth()`             Returns the depth of the built tree. |
| `int` | `getMaxInstancesInLeaf()`             Returns the maximum number of instances allowed in a leaf. |
| `double` | `getMaxRelativeLeafRadius()`             Returns the maximum relative radius of a leaf node. |
| `int` | `getNumLeaves()`             Returns the number of leaves in the built tree. |
| `int` | `getNumNodes()`             Returns the number of nodes (internal + leaf) in the built tree. |
| `java.lang.String[]` | `getOptions()`             Gets the current settings. |
| `java.lang.String` | `getRevision()`             Returns the revision string. |
| `java.util.Enumeration` | `listOptions()`             Returns an enumeration describing the available options. |
| `java.lang.String` | `maxInstancesInLeafTipText()`             Returns the tip text for this property. |
| `java.lang.String` | `maxRelativeLeafRadiusTipText()`             Returns the tip text for this property. |
| `void` | `setContainChildBalls(boolean containChildBalls)`             Sets whether if a parent ball should completely enclose its two child balls. |
| `void` | `setEuclideanDistanceFunction(EuclideanDistance func)`             Sets the distance function to use to build the tree. |
| `void` | `setInstanceList(int[] instList)`             Sets the master index array that points to instances in m\_Instances, so that only this array is manipulated, and m\_Instances is left untouched. |
| `void` | `setInstances(Instances inst)`             Sets the instances on which the tree is to be built. |
| `void` | `setMaxInstancesInLeaf(int num)`             Sets the maximum number of instances allowed in a leaf. |
| `void` | `setMaxRelativeLeafRadius(double radius)`             Sets the maximum relative radius, allowed for a leaf node. |
| `void` | `setOptions(java.lang.String[] options)`             Parses a given list of options. |

**Methods inherited from class java.lang.Object**

`equals, getClass, hashCode, notify, notifyAll, toString, wait, wait, wait`

**Constructor Detail**

### BallTreeConstructor

```
public BallTreeConstructor()
```

:   Creates a new instance of BallTreeConstructor.


**Method Detail**

### buildTree

```
public abstract BallNode buildTree()
                            throws java.lang.Exception
```

:   Builds the ball tree.

    :   **Returns:**: The root node of the tree. **Throws:**: `java.lang.Exception` - If there is problem building the tree.

---


### addInstance

```
public abstract int[] addInstance(BallNode node,
                                  Instance inst)
                           throws java.lang.Exception
```

:   Adds an instance to the ball tree.

    :   **Parameters:**: `node` - The root node of the tree.: `inst` - The instance to add to the tree. **Returns:**: The new master index array after adding the instance. **Throws:**: `java.lang.Exception` - If there is some problem adding the given instance to the tree.

---


### maxInstancesInLeafTipText

```
public java.lang.String maxInstancesInLeafTipText()
```

:   Returns the tip text for this property.

    :   **Returns:**: tip text for this property suitable for displaying in the explorer/experimenter gui.

---


### getMaxInstancesInLeaf

```
public int getMaxInstancesInLeaf()
```

:   Returns the maximum number of instances allowed in a leaf.

    :   **Returns:**: The maximum number of instances allowed in a leaf.

---


### setMaxInstancesInLeaf

```
public void setMaxInstancesInLeaf(int num)
                           throws java.lang.Exception
```

:   Sets the maximum number of instances allowed in a leaf.

    :   **Parameters:**: `num` - The maximum number of instances allowed in a leaf. **Throws:**: `java.lang.Exception` - If the num is < 1.

---


### maxRelativeLeafRadiusTipText

```
public java.lang.String maxRelativeLeafRadiusTipText()
```

:   Returns the tip text for this property.

    :   **Returns:**: tip text for this property suitable for displaying in the explorer/experimenter gui.

---


### getMaxRelativeLeafRadius

```
public double getMaxRelativeLeafRadius()
```

:   Returns the maximum relative radius of a leaf node.
    It is relative to the radius of the smallest ball enclosing all
    the data points (that were used to build the tree). This smallest
    ball would be the same as the root node's ball, if
    ContainChildBalls property is set to false (default).

    :   **Returns:**: The maximum relative radius allowed for a leaf.

---


### setMaxRelativeLeafRadius

```
public void setMaxRelativeLeafRadius(double radius)
                              throws java.lang.Exception
```

:   Sets the maximum relative radius, allowed for a leaf node. The
    radius is relative to the radius of the smallest ball enclosing all
    the data points (that were used to build the tree). This smallest
    ball would be the same as the root node's ball, if
    ContainChildBalls property is set to false (default).

    :   **Parameters:**: `radius` - The maximum relative radius allowed for a leaf. **Throws:**: `java.lang.Exception` - If radius is < 0.0.

---


### containChildBallsTipText

```
public java.lang.String containChildBallsTipText()
```

:   Returns the tip text for this property.

    :   **Returns:**: tip text for this property suitable for displaying in the explorer/experimenter gui.

---


### getContainChildBalls

```
public boolean getContainChildBalls()
```

:   Gets whether if a parent ball should completely enclose
    its two child balls.

    :   **Returns:**: true if parent ball is to enclose its child balls.

---


### setContainChildBalls

```
public void setContainChildBalls(boolean containChildBalls)
```

:   Sets whether if a parent ball should completely enclose
    its two child balls.

    :   **Parameters:**: `containChildBalls` - Should be tree if parent ball is to enclose its child balls.

---


### setInstances

```
public void setInstances(Instances inst)
```

:   Sets the instances on which the tree is to be built.

    :   **Parameters:**: `inst` - The instances on which to build the ball tree.

---


### setInstanceList

```
public void setInstanceList(int[] instList)
```

:   Sets the master index array that points to
    instances in m\_Instances, so that only this array
    is manipulated, and m\_Instances is left
    untouched.

    :   **Parameters:**: `instList` - The master index array.

---


### setEuclideanDistanceFunction

```
public void setEuclideanDistanceFunction(EuclideanDistance func)
```

:   Sets the distance function to use to build the
    tree.

    :   **Parameters:**: `func` - The distance function.

---


### getNumNodes

```
public int getNumNodes()
```

:   Returns the number of nodes (internal + leaf)
    in the built tree.

    :   **Returns:**: The number of nodes in the tree.

---


### getNumLeaves

```
public int getNumLeaves()
```

:   Returns the number of leaves in the built tree.

    :   **Returns:**: The number of leaves in the tree.

---


### getMaxDepth

```
public int getMaxDepth()
```

:   Returns the depth of the built tree.

    :   **Returns:**: The depth of the tree.

---


### listOptions

```
public java.util.Enumeration listOptions()
```

:   Returns an enumeration describing the available options.

    :   **Specified by:**: `listOptions` in interface `OptionHandler`
    :   **Returns:**: an enumeration of all the available options.

---


### setOptions

```
public void setOptions(java.lang.String[] options)
                throws java.lang.Exception
```

:   Parses a given list of options.

    :   **Specified by:**: `setOptions` in interface `OptionHandler`
    :   **Parameters:**: `options` - the list of options as an array of strings **Throws:**: `java.lang.Exception` - if an option is not supported

---


### getOptions

```
public java.lang.String[] getOptions()
```

:   Gets the current settings.

    :   **Specified by:**: `getOptions` in interface `OptionHandler`
    :   **Returns:**: an array of strings suitable for passing to setOptions

---


### getRevision

```
public java.lang.String getRevision()
```

:   Returns the revision string.

    :   **Specified by:**: `getRevision` in interface `RevisionHandler`
    :   **Returns:**: the revision


---


|  |  |  |
| --- | --- | --- |
| **Overview** \| **Package** \| **Class** \| **Tree** \| **Deprecated** \| **Index** \| **Help** \| **Weka's home** | |  |
| **PREV CLASS**   **NEXT CLASS** | **FRAMES**    **NO FRAMES**     **All Classes** |  |
| SUMMARY: NESTED \| FIELD \| CONSTR \| METHOD | DETAIL: FIELD \| CONSTR \| METHOD |  |


---
